# Involvement of the endocannabinoid system in the physiological response to transient common carotid artery occlusion and reperfusion

**DOI:** 10.1186/s12944-016-0389-y

**Published:** 2017-01-19

**Authors:** Marina Quartu, Laura Poddighe, Tiziana Melis, Maria Pina Serra, Marianna Boi, Sara Lisai, Gianfranca Carta, Elisabetta Murru, Laura Muredda, Maria Collu, Sebastiano Banni

**Affiliations:** Department of Biomedical Sciences, Cittadella Universitaria di Monserrato, 09042 Monserrato, CA Italy

**Keywords:** Bilateral common carotid artery occlusion, Reperfusion, Endocannabinoid system, Lipoperoxides, COX-2, Cerebral cortex

## Abstract

**Background:**

The transient global cerebral hypoperfusion/reperfusion achieved by induction of Bilateral Common Carotid Artery Occlusion followed by Reperfusion (BCCAO/R) may trigger a physiological response in an attempt to preserve tissue and function integrity. There are several candidate molecules among which the endocannabinoid system (ECS) and/or peroxisome-proliferator activated receptor-alpha (PPAR-alpha) may play a role in modulating oxidative stress and inflammation. The aims of the present study are to evaluate whether the ECS, the enzyme cyclooxygenase-2 (COX-2) and PPAR-alpha are involved during BCCAO/R in rat brain, and to identify possible markers of the ongoing BCCAO/R-induced challenge in plasma.

**Methods:**

Adult Wistar rats underwent BCCAO/R with 30 min hypoperfusion followed by 60 min reperfusion. The frontal and temporal-occipital cortices and plasma were analyzed by high performance liquid chromatography-mass spectrometry (HPLC-MS) to determine concentrations of endocannabinoids (eCBs) and related molecules behaving as ligands of PPAR-alpha, and of oxidative-stress markers such as lipoperoxides, while Western Blot and immunohistochemistry were used to study protein expression of cannabinoid receptors, COX-2 and PPAR-alpha. Unpaired Student’s *t*-test was used to evaluate statistical differences between groups.

**Results:**

The acute BCCAO/R procedure is followed by increased brain tissue levels of the eCBs 2-arachidonoylglycerol and anandamide, palmitoylethanolamide, an avid ligand of PPAR-alpha, lipoperoxides, type 1 (CB1) and type 2 (CB2) cannabinoid receptors, and COX-2, and decreased brain tissue concentrations of docosahexaenoic acid (DHA), one of the major targets of lipid peroxidation. In plasma, increased levels of anandamide and lipoperoxides were observed.

**Conclusions:**

The BCCAO/R stimulated early molecular changes that can be easily traced in brain tissue and plasma, and that are indicative of the tissue physiological response to the reperfusion-induced oxidative stress and inflammation. The observed variations suggest that the positive modulation of the ECS and the increase of proinflammatory substances are directly correlated events. Increase of plasmatic levels of anandamide and lipoperoxides further suggests that dysregulation of these molecules may be taken as an indicator of an ongoing hypoperfusion/reperfusion challenge.

## Background

The transient Bilateral Common Carotid Artery Occlusion followed by Reperfusion (BCCAO/R) is a model of transient global hypoperfusion [1]. This model does not lead to an authentic ischemic insult in the rodent brain due to the presence of efficient collateral systems, which allow for a cerebral blood flow compensation within a few minutes [2]. However, it has been recently shown that, in the rat, the cerebral transient hypoperfusion followed by reperfusion, such as that induced by the BCCAO/R, causes detectable and consistent tissue changes such as a decrease of docosahexaenoic acid (DHA), one of the most abundant polyunsaturated fatty acid in neuronal membrane phospholipids, an increase of the cyclooxygenase-2 (COX-2) [3] and significant microvascular alterations [4].

The endocannabinoids (eCBs) are endogenous lipid mediators involved in a wide variety of biological processes. The most frequently studied eCBs are N-arachidonoyl-ethanolamide (AEA, anandamide), belonging to the superfamily of N-acylethanolamides (NAEs), and 2-arachidonoyl-glycerol (2-AG). AEA and 2-AG are not stored in vesicles but they are synthesized ‘on demand’ and released from plasmatic membranes immediately after their production when cells are challenged with potentially harmful stimuli [5–7]. Accumulating data show that eCBs, with their signaling-mediating receptors, and enzymes involved in their synthesis and degradation, constitute an extensive system with relative basic levels of each component that is modulated by different and concurring molecular mechanisms [8, 9]. Growing evidence supports a general role for eCBs and congeners in the preservation of metabolic homeostasis and responsivity of the brain to stress, and indicate that they are involved in inflammation and act as endogenous neuroprotectants in cerebral ischemia [5, 9–18]. As for the likely involvement of eCB in neuroinflammation, it has been shown that 2-AG and AEA are substrates for COX-2 [19] and it is relevant that the eCB neuroprotective activity may be mediated by preventing excessive expression of COX-2 [11, 20, 21]. Conversely, COX-2 is a key regulator of eCB signalling [22]. Moreover, it has been shown that the peroxisome-proliferator activated receptor (PPAR)-alpha mediates the anti-inflammatory effects of the non-cannabinoid palmitoylethanolamide (PEA) that behaves as one of its endogenous ligands [23]. Though the role of eCB system in neuroprotection is somewhat controversial, data on focal cerebral ischemia followed by reperfusion show that i) intraperitoneally administered PEA and AEA reduce the size of infarcted tissue [7], ii) both pharmacological blockade [24] and genetic deletion of CB1 receptor reduce the infarct volume and improve neurological function [25], and iii) CB2 activation attenuates the cerebral ischemia/reperfusion-induced microcirculatory dysfunction [13].

In this study, we investigated whether the eCB system may be directly correlated to the oxidative events triggered by the BCCAO/R-induced transient global hypoperfusion in rat brain, and we chose to examine the forebrain areas that are directly and selectively reached by the internal carotid artery branches. With this aim, we examined, prior to and after induction of BCCAO/R, cerebral tissue and plasmatic concentrations of eCBs and of oxidative stress markers such as polyunsaturated fatty acid (PUFA) hydroperoxides. Parallel immunochemical analyses, carried out by means of Western Blot and immunohistochemistry, investigated possible BCCAO/R-induced changes in the occurrence and tissue distribution of CB1 and CB2 receptors, COX-2, and the transcription factor peroxisome-proliferator activated receptor (PPAR)-alpha. Results are discussed in view of the possible use of eCBs and lipoperoxides as markers of an ongoing transient cerebral global hypoperfusion.

## Methods

### Experimental procedure

#### Animals and keeping

For 1 week before the start of the experiment, fifty six adult male Wistar rats (Harlan-Italy, Udine, Italy), weighing 210 ± 20 g (mean ± SD) were housed under controlled temperature (21 ± 2 °C), relative humidity (60 ± 5%) and artificial 12 h light/dark cycle, avoiding all stressful stimuli. Animal handling and care throughout the experimental procedures met with national (Legislative Decree n. 26, 04/04/2014) and international (Directive 2010/63/EU in Europe) laws and policies. The experimental protocols were carried out in compliance with the guidelines of the Animal Ethics Committee of the University of Cagliari. Standard laboratory food (A04, Safe, Augy, France) and water were freely available *ad libitum*.

According to the optimum standard for the evaluation of lipids in tissue and plasma [6, 26], animals received no food for 12 h before surgery. Rats were randomly assigned to two groups (*n* = 12/group): one group was submitted to BCCAO and reperfused (BCCAO/R) and one group was sham-operated. Surgery was performed in all cases between 13:00 and 16:30 p.m..

#### Surgery

Surgical procedure for induction of BCCAO/R was adapted from the method of Iwasaki et al. [27] and performed in all cases between 13:00 and 16:30 p.m.. Rats were anesthetized with intraperitoneal administration of Equitesin (4.2% w/v chloral hydrate, 2.12% w/v MgSO_4_, 16.2% w/w pentobarbital, 39.6% w/w propylene glycol, and 10% w/w ethanol in sterile distilled H_2_O) (0.5 ml/100 g bodyweight). After a midline cervical incision and blunt dissection of muscles, the common carotid arteries (CCA) were exposed while leaving the vagus nerve intact. Cerebral blood flow reduction was produced by placement of two atraumatic microvascular clips for 30 min on CCA. The reperfusion period was achieved by removing the clips and restoring blood flow through the stenosed vessels for 60 min. The control animals, used to determine the effects of anaesthesia and surgical manipulation of the results, were represented by sham-operated rats that underwent surgery without CCA occlusion.

### Sampling

At the end of the procedure, brain samples were collected either as fresh tissue for lipid analysis and Western Blot or after transcardial perfusion fixation with ice cold 4% formaldehyde in 0.1 M phosphate buffer (PB), pH 7.4 for immunohistochemistry. The frontal cortex was rapidly dissected out by a transverse cut made at the level of the optic chiasm, at the approximate bregma level of −1.0 mm [28], and frozen at −80 °C until HPLC or Western Blot analysis. Temporal-occipital cortex, dissected out by a transverse cut at the approximate bregma level of −4.5 mm, was also sampled as a control cortical area not irrorated by the internal carotid artery branches. Blood was quickly collected from the trunk of killed animals into heparinised tubes and centrifuged at 1500 g for 10 min at −8 °C. The resulting plasma was frozen at −20 °C until assayed for lipids. Immunohistochemistry was used to determine the tissue distribution and cellular localization of the same markers used for the Western Blot analysis. Perfused brains were dissected out and then rinsed overnight in 0.1 M PB, pH 7.3, containing 20% sucrose. After sucrose infiltration, samples were embedded in Optimal Cutting Temperature (OCT) medium for cryostat sectioning. For each assay, the investigator was blind with respect to the experimental condition of the rats.

### Endocannabinoid and congener quantifications

**Fig. 1:**
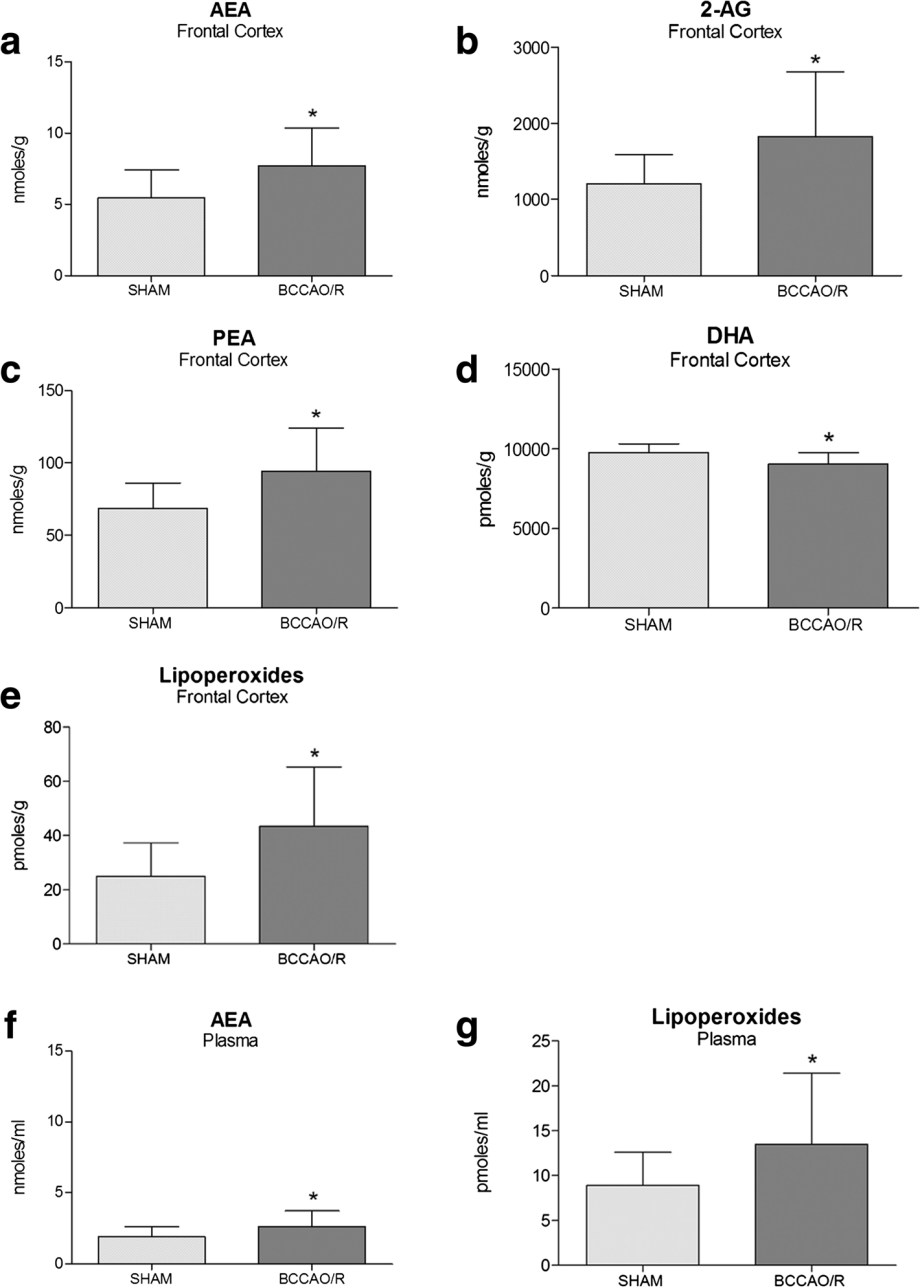
Endocannabinoids and fatty acid profile in frontal cortex and plasma. High performance liquid chromatography (HPLC) analysis of frontal cortex (**a**−**f**) and plasma (**g**, **h**) of sham-operated and bilateral common carotid artery occlusion followed by reperfusion (BCCAO/R) rats. N-arachidonoyl-ethanolamide (AEA) (**a**, **e**), 2-arachidonoyl-glycerol (2-AG) (**b**), palmitoylethanolamide (PEA) (**c**), docosahexaenoic acid (DHA) (**e**) and lipoperoxides (**f**, **h**) concentrations are reported as mean values of 12 sham-operated and 12 BCCAO/R rats. Error bars depict S.D.. Unpaired Student’s *t* test: *p < 0.05

Frozen tissues were homogenized and extracted with 50 mM chloroform/methanol/Tris–HCl, pH 7.5 (2:1:1, v/v), containing internal deuterated standards for anandamide (AEA), 2-arachidonoyl-monoacylglycerol (2-AG), palmitoylethanolamide (PEA) and oleoylethanolamide (OEA) quantification by isotope dilution ([2H]^8^ AEA, [2H]^5 ^2-AG, [2H]^4^ PEA, [2H]^4^ OEA; Cayman Chemical, Ann Arbor, MI, USA). AEA, 2-AG, PEA, and OEA were quantified by liquid chromatography–atmospheric pressure chemical ionization–mass spectrometry [1100 HPLC system (Agilent Technologies, Santa Clara, CA, USA) equipped with MS Detector 6110 single quadrupole] and using selected ion monitoring at M1 values for the four compounds and their deuterated homologs, as described previously [26]. Concentrations (nmoles/g; nmoles/ml) are shown as histograms in Fig. 1.

### Tissue fatty acid quantifications

Total lipids were extracted from different brain areas using chloroform/methanol 2:1 (v/v). Aliquots were mildly saponified as previously described [29] in order to obtain free fatty acids for high-performance liquid chromatography (HPLC) analysis. Separation of fatty acids was carried out with an Agilent 1100 HPLC system (Agilent Technologies) equipped with a diode array detector as previously reported [6]. Concentrations (nmoles/g; nmoles/ml) are shown as histograms in Fig. 1.

### Western blot

**Fig. 2:**
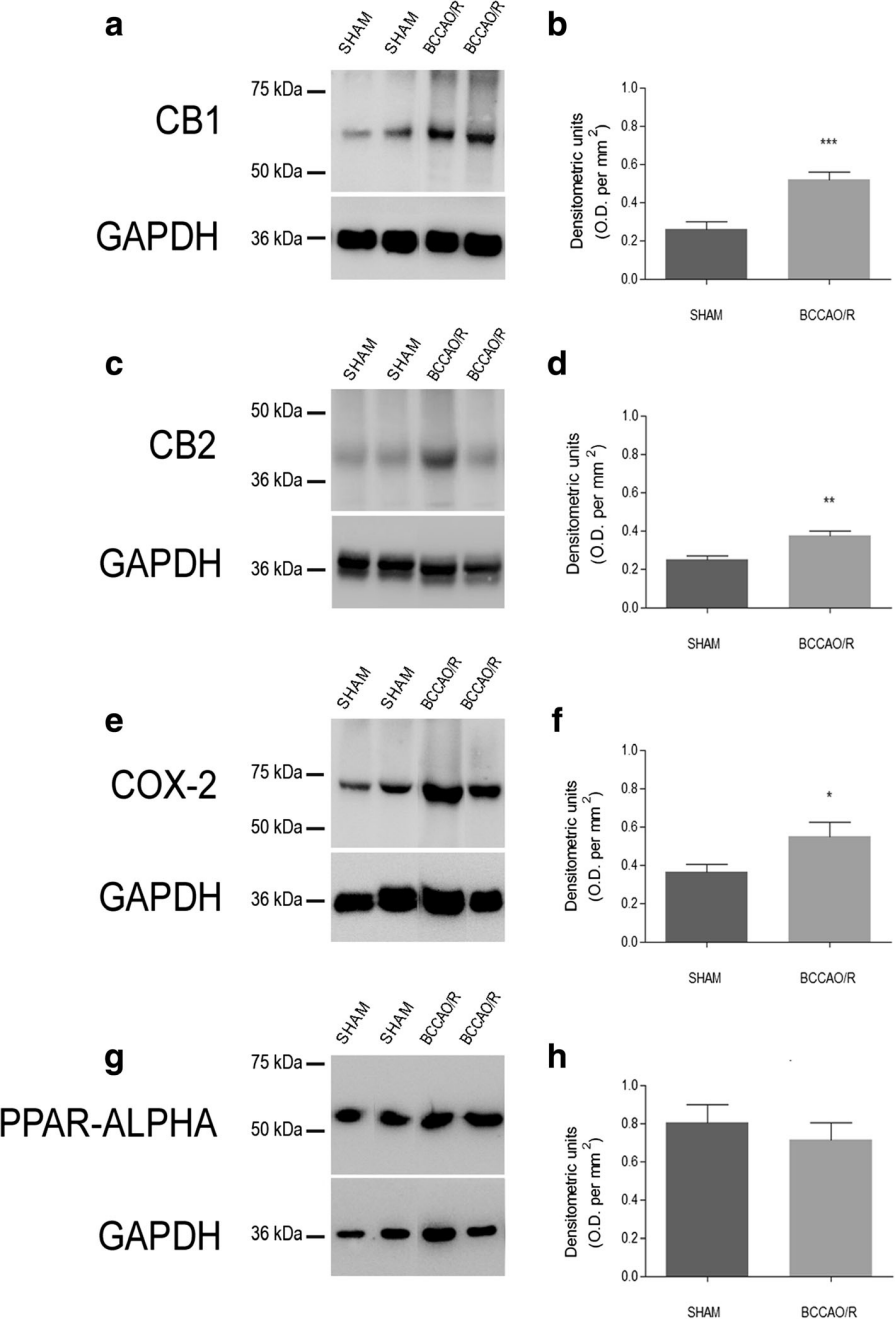
Western Blot analysis. Cannabinoid receptors CB1 (**a**, **b**) and CB2 (**c**, **d**), cyclooxygenase-2 (COX-2) (**e**, **f**) and peroxisome-proliferator activated receptor-alpha (PPAR-alpha) (**g**, **h**) in the frontal cortex of sham and bilateral common carotid artery occlusion followed by reperfusion (BCCAO/R) rats. **b**, **d**, **f**, **h**: densitometric analysis of the band gray levels expressed as a percentage of the optical density (O.D.) ratio of immunostained bands to those of GAPDH. Data are reported as mean values of 12 sham-operated and 12 BCCAO/R rats. Error bars depict S.D. Unpaired Student’s *t* test: *p < 0.05

Tissue homogenates were prepared in a 2% solution of sodium dodecyl sulfate (SDS) containing a cocktail of protease inhibitors (cOmplete, Mini Protease Inhibitor Cocktail Tablets, Roche, Basel, Switzerland). Protein concentrations were determined using the Lowry method of protein assay [30] with bovine serum albumin as standard. Proteins for each tissue homogenate (40 μg), diluted 3:1 in 4× loading buffer (NuPAGE LDS Sample Buffer 4×, Novex by Life Technologies, Carlsbad, CA, USA), were heated to 95 °C for 10 min and separated by SDS-polyacrilamide gel electrophoresis (SDS-PAGE) using precast polyacrylamide gradient gel (NuPAGE 4–12% Bis-Tris Gel Midi, Novex by Life Technologies) in the XCell4 Sure Lock™ Midi-Cell chamber (Life Technologies). Internal mw standards (Precision Plus Protein™ WesternC™ Standards, Bio-Rad, Hercules, CA, USA) were run in parallel. Two gels at a time were run for Coomassie staining and immunoblotting, respectively. Proteins for immunoblotting were electrophoretically transferred on a polyvinylidene fluoride membrane (Amersham Hybond™-P, GE Healthcare, Little Chalfont, United Kingdom) using the Criterion™ Blotter (Bio-Rad). Blots were blocked by immersion in 20 mM Tris base and 137 mM sodium chloride (TBS) containing 5% milk powder and 0.1% Tween 20 (TBS-T), for 60 min at room temperature and incubated overnight at 4 °C with rabbit polyclonal antisera directed against CB1 receptor (Synaptic System, Göttingen, Germany), diluted 1:500, CB2 receptor (MyBioSource, San Diego, CA, USA), diluted 1:1000, COX-2 (residues 570–598) (Cayman Chem.), diluted 1:200, and PPAR-α (Thermo Scientific, Waltham, MA, USA), diluted 1:1000, in TBS containing 5% milk powder and 0.02% sodium azide, were used as primary antisera. After TBS-T rinse, blots were incubated for 60 min, at room temperature, with peroxidase-conjugated goat anti-rabbit serum (Sigma Aldrich, Saint Louis, MO, USA), diluted 1:10,000 in TBS/T. Loading controls were obtained by stripping and immunostaining the membranes with a mouse monoclonal antibody against the housekeeping protein glyceraldehyde 3-phosphate dehydrogenase (GAPDH) (EMD Millipore, Darmstadt, Germany), diluted 1:1000, as primary antiserum, and a peroxidase-conjugated goat anti-mouse serum (EMD Millipore), diluted 1:5000, as secondary antiserum. In order to control for non specific staining, blots were stripped and incubated with the relevant secondary antiserum. After TBS-T rinse, protein bands were visualized using the ECL chemiluminescent system according to the protocol provided by the company (GE Healthcare), under ImageQuant LAS 4000. Approximate molecular weight (mw) and relative optical density (O.D.) of immunolabelled protein bands were evaluated by a “blind” examiner, and were quantified by comparing the position of relevant bands on the digital images with those of the GAPDH bands, respectively. The ratio of the intensity of COX-2- and PPAR-α-positive bands to the intensity of GAPDH-positive ones was used to compare relative expression levels of these proteins following BCCAO/R procedure. O.D. was quantified by ImageJ (http://imagej.nih.gov/ij/). Concentrations (nmoles/g; nmoles/ml) are shown as histograms in Fig. 2.

### Immunohistochemistry

The avidin–biotin–peroxidase complex (ABC) and the indirect immunofluorescence techniques were used to process cryostat semiconsecutive sections (16 μm thick), collected on chrome alum-gelatin coated slides. Coronal serial sections at + 4.70 to - 1.70 mm and at - 3.5 to - 8.0 mm Bregma levels, respectively, were used to focus the observations on the frontal and temporal-occipital cortex [28]. For the ABC, the endogenous peroxidase activity was blocked with 0.1% phenylhydrazine in phosphate buffered saline (PBS) containing 0.2% Triton X-100 (PBS/T), followed by incubation with 20% of either normal goat or normal horse serum (Vector, Burlingame, CA, USA) for 1 h at RT. For ABC, the primary antibodies were rabbit polyclonal antibody against CB1 (Synaptic System), diluted 1:1000 and COX-2 (Cayman Chem.), diluted 1:300. For immunofluorescence, the primary antibodies were goat polyclonal antiserum against COX-2 (SantaCruz Biotechnology), diluted 1:100, mouse monoclonal antibody against Iba1 (WAKO), diluted 1:1000, rabbit polyclonal antiserum against Glial Fibrillary Acidic Protein (GFAP) (DAKO), diluted 1:1000. Incubations with primary antiserum were carried out overnight at 4 °C. Biotin-conjugated goat anti-rabbit serum (Vector, Burlingame, CA, USA), diluted 1:400, was used as secondary antiserum in the ABC method; Alexa Fluor 488 or 594 donkey anti-goat, anti-mouse and anti-rabbit sera (Invitrogen, Eugene, OR, USA), diluted 1:500, were used as secondary antiserum in the immunofluorescence technique. The ABC reaction product was revealed with a biotin-conjugated goat anti-rabbit serum (Vector, Burlingame, CA, USA), diluted 1:400, as secondary antiserum. The ABC (BioSpa Div. Milan, Italy), diluted 1:250, followed by a solution of 0.1 M PB, pH 7.3, containing 0.05% 3,3’-diaminobenzidine (Sigma Aldrich), 0.01% hydrogen peroxide and 0.04% nickel ammonium sulfate were used to reveal the reaction product. Incubations with secondary antiserum and ABC lasted 60 min and were performed at RT. Negative control preparations were obtained by incubating tissue sections in parallel with either PBS-T alone or with the relevant primary antiserum preabsorbed with an excess of the corresponding peptide antigen. Slides were observed by the same examiner, who was “blind” with respect to the animals’ treatment, with an Olympus BX61 microscope, equipped with epifluorescence illumination, and digital images (Figs. 4, 5) were captured with a Leica DF 450C camera.

### Statistical analysis

Data from the two experimental groups, sham-operated animals and the BCCAO/R ones, are depicted in the figures as mean ± standard deviation (S.D.) and statistical differences were determined by unpaired Student’s *t*-test.

## Results

### eCB and fatty acid profiles in brain tissue

In the frontal cortex AEA, 2-AG, and PEA were found significantly increased in BCCAO/R rats as compared to the sham-operated ones (*p* < 0.05) by 41, 51 and 37%, respectively (Fig. 1a–c), while DHA decreased by about 7% (*p* < 0.001) (Fig. 1d), and lipoperoxides, considered as a molecular marker of oxidative stress, increased by 57% (*p* < 0.05) (Fig. 1e). No statistically significant changes were observed in the temporal-occipital cortex (data not shown).

### eCB and fatty acid profiles in plasma

In BCCAO/R rats, analysis of eCBs and congeners revealed that levels of AEA increased significantly by about 38% compared to sham rats (*p* < 0.05) (Fig. 1f), whereas no change was observed in 2-AG, PEA, and OEA levels. A strong increase of lipoperoxides could also be detected (52%; *p* < 0.05) (Fig. 1g). No statistically significant changes were observed in the temporal-occipital cortex (data not shown).

### Western blot

After the BCCAO/R, protein changes were detected only in the frontal cortex homogenates, whereas the temporal-occipital cortex appeared unaffected. Thus, the relative levels of the CB1 receptor protein increased by 101% (*p* < 0.0001) (Fig. 2a, b), that of CB2 by 51% (*p* < 0.05) (Fig. 2c, d) and that of COX-2 by 50% (Fig. 2e, f) (*p* < 0.05). No changes were detectable for the relative levels of PPAR-α (Fig. 2g, h). The antibodies against CB1 and COX-2 were also the only ones to produce a reliable immunostaining in tissue sections of rat cerebral cortex. For this reason, the following immunohistochemical data are based exclusively on the immunoreactivity that they provided.

### Immunohistochemistry

**Fig. 3:**
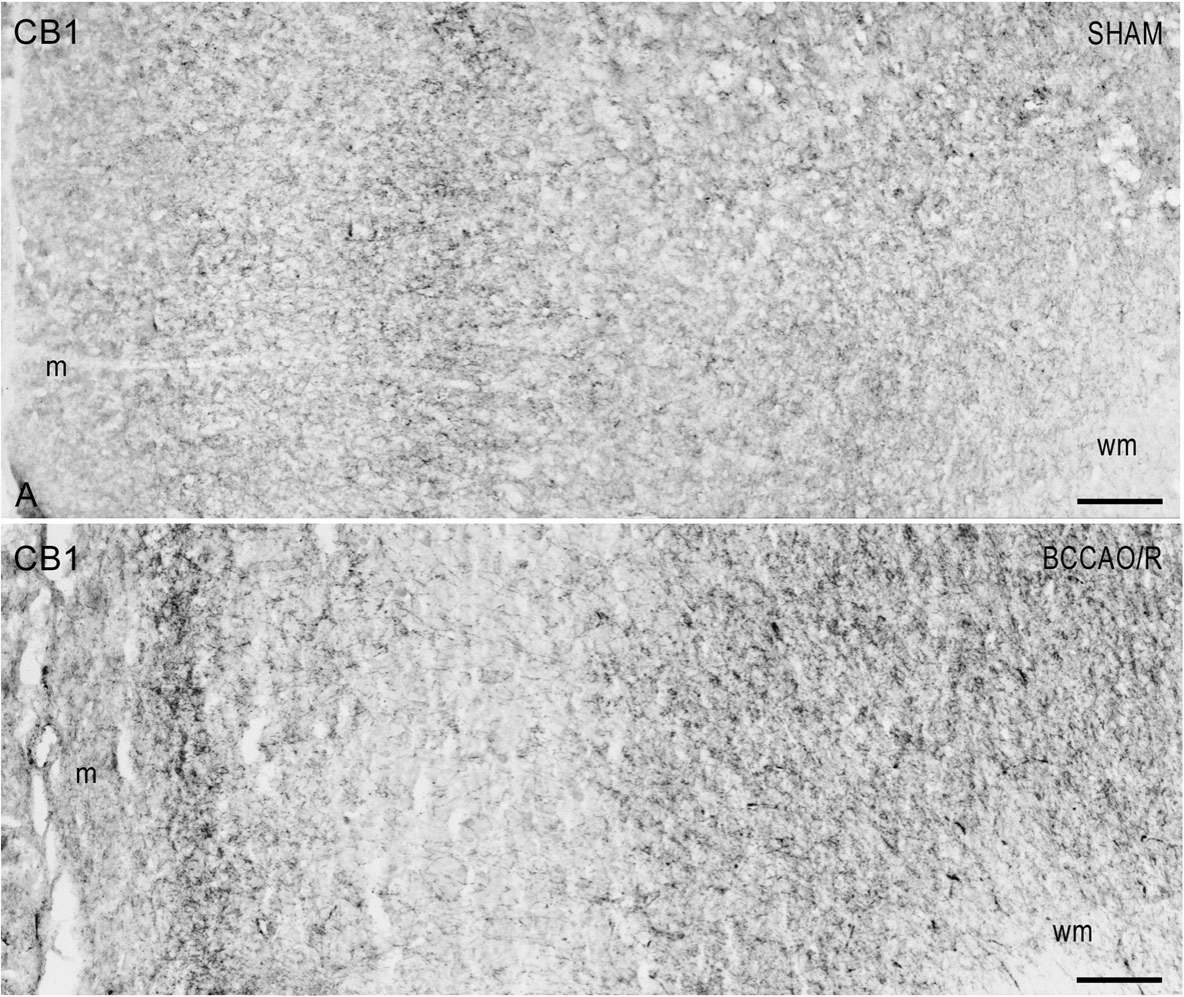
CB1-like immunoreactivity in the frontal cortex. Brain sections of sham-operated and BCCAO/R rats are shown in (**a**) and (**b**), respectively. Panels (**a**) and (**b**) are representative of observations carried out in 6 sham-operated and 6 BCCAO/R rats. Positive nervous structures are distributed throughout the cortical layers. m, molecular layer; wm, white matter. Scale bars: 50 μm

**Fig. 4:**
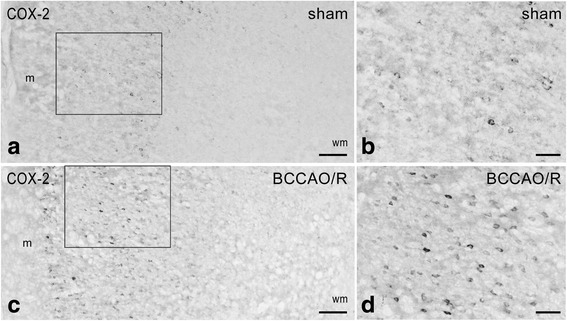
Cyclooxygenase-2 (COX-2)-like immunoreactivity in the frontal cortex. Brain sections of sham-operated and BCCAO/R rats are shown in (**a**, **b**) and (**c**, **d**), respectively. **b**, **d** represent a higher magnification of the microscopic fields *squared* in (**a**) and (**c**), respectively. Panels (**a**–**d**) are representative of observations carried out in 6 sham-operated and 6 BCCAO/R rats. Positive cell bodies are mostly distributed in the superficial cortical layers. m, molecular layer; wm, white matter. Scale bars: 50 μm

In order to find a possible association between the molecular changes observed by HPLC and Western Blot analyses and the tissue morphology, immunoreactivities to CB1 and COX-2 were also examined in the cerebral cortex. All markers labelled neuronal structures distributed throughout the rostro-caudal extension of the frontal cortex (Figs 3 and 4) and the temporal-occipital cortex (data not shown).

**Fig. 5:**
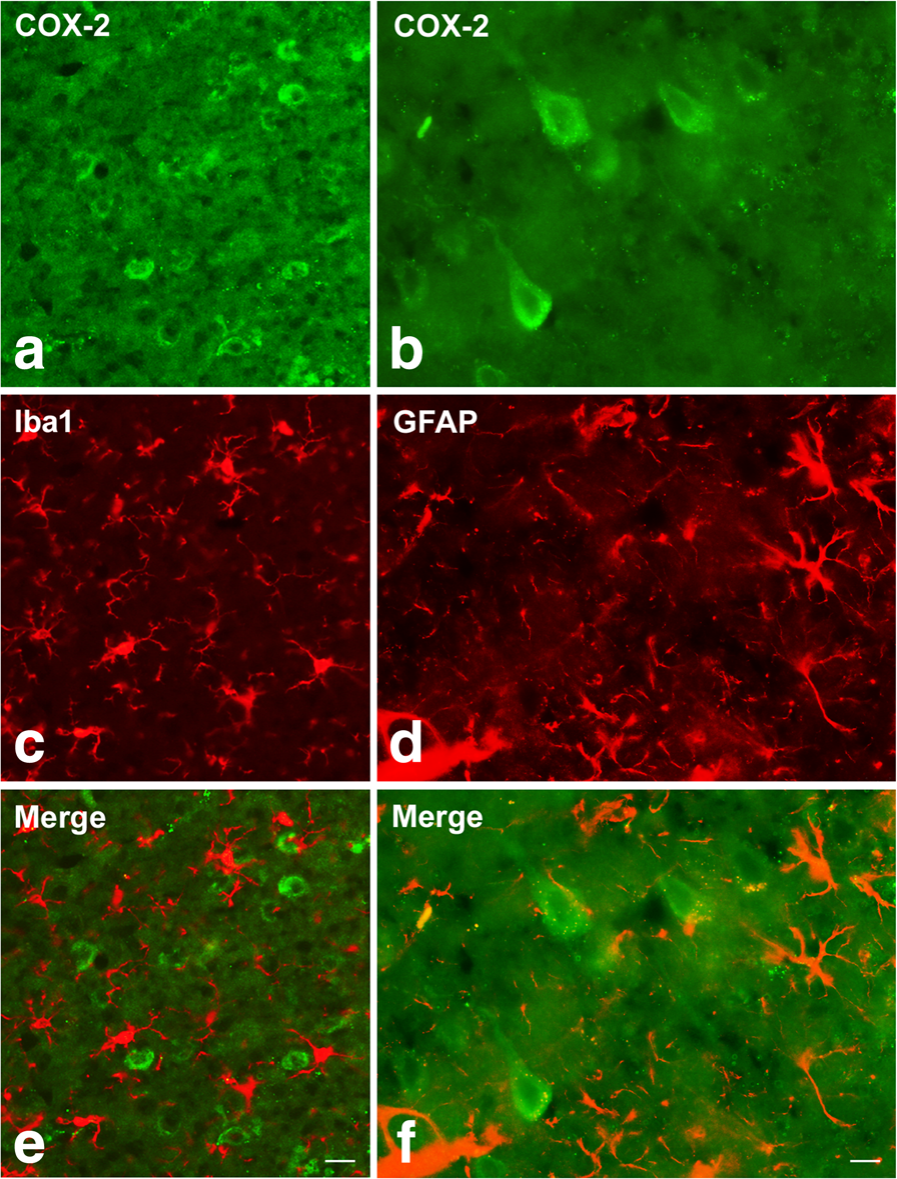
Double immunofluorescence for cyclooxygenase-2 (COX-2) (**a**, **b**, **e**, **f**) and either Iba1 (**c**, **e**) or glial fibrillary acidic protein (GFAP) (**d**, **f**) in the frontal cortex of BCCAO/R rats. Scale bars E = A, C: 25 μm; F = B, D: 10 μm

CB1 receptor-antibody labelled a dense to moderate plexus of beaded fibers and a discrete number of neuronal cell bodies distributed throughout the cortical layers (Fig. 3). As a general rule the density of labelled nerve terminals and fibers appeared higher in BCCAO/R animals (Fig. 3b) than in the sham-operated ones (Fig. 3a). By contrast, the COX-2-like immunoreactivity appeared as intracytoplasmic granules of different density in the perikaryon and proximal processes of neuronal cells (Fig. 4), being intensely stained and easily detectable in layers II/III and V. As a general rule, the staining intensity and density of labelled structures were higher in BCCAO/R (Fig. 4b) than in sham-operated rat brains (Fig. 4a). Representative double immunostainings for COX-2 and either glial marker Iba1 (for microglia) or GFAP (for astrocytes), carried out in the BCCAO/R rats, showed the absence of colocalization and demonstrated that COX-2 immunolabelling was localized to neuronal cell bodies (Fig. 5).

## Discussion

The primary findings of this study are that BCCAO for 30 min followed by 60 min reperfusion of the brain is sufficient to cause molecular changes including: i) a significant positive modulation of the eCB system, involving the cerebral tissue and plasmatic levels of eCBs and congeners and tissue expression of the CB1 receptor; ii) a decrease of DHA tissue levels; iii) an increase of lipoperoxide levels; iv) a parallel increase of COX-2 expression in frontal cortex suggesting that they are part of a physiological response to the hypoperfusion/reperfusion insult.

### Modulation of the eCB system by hypoperfusion/reperfusion

According to previous studies [12, 31, 32], our data point out that in cerebral cortex there are basal differences in the occurrence and relative concentrations of eCBs, the tissue content of which is increased after BCCAO/R. This last observation is in line with studies showing that brain focal ischemia and reperfusion activate the eCB system by driving a combination of biochemical adaptations of the NAE degrading and synthesizing enzymes that, collectively, lead to the accumulation of eCBs [12, 33]. The eCB congener PEA is an important player in the endogenous defense against neuroinflammation [33] and has beneficial pharmacological effects in several animal models of inflammation [11, 33–35]. In particular, PEA may exert neuroprotective effects by modulating the immune cells [36], activating PPAR-alpha [23, 37] and blunting COX-2 activity [11]. Exogenous administration of PEA in an acute stroke model is effective in reducing the infarct size [7]. Moreover, in human stroke eCBs and congeners have been shown to become detectable during the acute phase of an ischemic stroke where they may play a role through multiple potential mechanisms [17]. Research into this suggested that the inhibition or modulation of the enzymatic breakdown of PEA may represent a complementary therapeutic approach to counteract neuroinflammation [33].

Our data also show that after BCCAO/R a concomitant 38% increase of AEA could be detected in plasma. It is difficult to speculate whether the eCB modifications in brain tissue are directly correlated to the observed increase in AEA plasma levels. Clinical studies support the notion that neurological and neuropsychiatric disorders are characterized by detectable changes in eCB plasma levels [17 and refs therein]. Raise in the eCB levels in acute stroke patients have been detected by in vivo microdialysis [5] and plasma analysis [17]. Interestingly, the increases of AEA and PEA plasma level have been reported to show a positive correlation with infarct volume and/or neurological disability, so that patients with higher AEA and PEA levels had greater neurological impairment [17]. It has been proposed that a contribution to eCB and congener plasma levels might be due to a brain damage “spill over” effect to the peripheral circulation, the extent of which may reflect the severity of central nervous system pathology [14, 17]. On the other hand, it has been reported that the BCCAO modifies the pial microvasculature and that restoration of blood circulation in reperfusion causes a reactive hyperaemia [4]. Whether similar changes in vascular tone are responsible for setting off or modulating the brain “spill over” of eCBs into systemic circulation is an important issue to be taken into consideration. As for the functional meaning of the increase in eCB levels following the BCCAO/R, it can be inferred that it contributes to neuroprotection by different pathways. Whether in the BCCAO/R model used in this study the apparently concomitant raise in eCB concentrations and COX-2 protein levels are mutually dependent events is an issue that remains to be clarified [38].

In this study, we have also demonstrated that, after BCCAO/R, higher concentration of eCBs are associated with an increase of the relative levels of CB1 and CB2 receptor proteins. We may speculate that CB1 may act synergistically to increase eCB tone, which may be critical for protection against transient hypoperfusion/reperfusion cerebral insult [25]. Moreover, in agreement with previous observations [39], the extensive CB1-positive innervation that we observed in the cerebral cortex may suggest that intrinsic local circuits may contribute to the modulation of the reperfusion-induced tissue challenge. It is tempting to hypothesize that the BCCAO/R-induced increase of relative CB2 protein levels adds to this framework, probably regulating the production of pro-inflammatory molecules by glial cells, through which CB2 may either prevent the detrimental effects of neuroinflammatory reaction or participate in adaptive changes to the brain insult [40, 41]. As already proposed [42, 43], a parallel increase of the levels of eCBs and CB1 receptors indicates a sensitization of the cannabinoidergic system that may contribute to the regulation of cellular functions that depend upon CB1 receptor activation. It can be suggested that, after BCCAO/R, this sensitization modulates events such as neurotransmitter release, calcium cellular influx, oxidative stress damage, and vascular tone that appear to be crucial in the response of cerebral tissue to the ischemia/reperfusion insult. Further, in light of the evidence that eCBs may act as ligands for receptors other than CBs [44], it is interesting that the endocannabinoid and endovanilloid pathways have been found to antagonistically interact to adjust synaptic strength of inhibitory synapses [45].

### Evidences for hypoperfusion/reperfusion-induced oxidative stress

In line with our previous findings [3], the present data indicate that the BCCAO/R causes a significant decrease of DHA, a polyunsaturated fatty acid that is richly endowed and avidly retained in the brain [46, 47] and confirm that the BCCAO/R model is sufficient to perturb tissue homeostasis by disrupting the normal DHA levels and thus by potentially affecting the efficiency of membrane-depending molecular mechanisms [48]. DHA is recognized to be particularly liable to lipid peroxidation [49] and, therefore, to be potentially apt to contribute to the hypoperfusion/reperfusion-induced oxidative stress. However, several lines of evidence have associated increased levels of DHA with tissue protection in neuroinflammation [49], suggesting that DHA *per se* does not increase susceptibility to oxidative stress. Data in rodents and healthy humans support this inference, as dietary supplementation with n-3 PUFAs does not affect lipid peroxidation [50, 51]. By contrast, since DHA has been shown to have a role in neuroprotection after brain hypoxia and ischemia, it is relevant that recent prophylactic and therapeutic approaches for cerebrovascular disease take into account the pathways of brain accretion and delivery of DHA [48, 52].

In our study, eCB changes were further characterized by a marked increase of tissue and plasmatic concentrations of lipoperoxides that is one of the hallmarks of the reperfusion-induced oxidative stress [53, 54]. Lipoperoxides are quite unstable compounds that are capable of extending the free radical oxidative damage, and forming proinflammatory substances [53, 54]. The increase in lipoperoxide levels without any evident histological alteration of cerebral tissue is in line with previous experimental findings on a rat model of BCCAO/R similar to ours [55].

### Clinical implications

Increase of levels of lipoperoxides and a concomitant increase of their catabolism in peroxisomes have been shown to be directly correlated with the hypoperfusion/reperfusion-induced oxidative challenge in patients undergoing carotid endoarterectomy [55]. In particular, it is interesting that peroxisomal beta-oxidation increased during the first 30 min of reperfusion only in patients having contralateral carotid stenosis higher than 50% to decrease thereafter within 2 h from reperfusion [55].

Several physiopathological conditions, not necessarily associated with early obvious neurological signs [56], share the occurrence of cerebral hypoperfusion episodes for which the detection of molecular indicators in the early hours may be useful in clinical settings to prevent irreversible cerebral damage. The question of whether plasmatic changes of AEA and lipoperoxides could represent additional specific markers in humans should be further investigated.

## Conclusions

The present study showed that the 30/60 min BCCAO/R procedure activates the ECS in rat and induces parallel changes in the fatty acid tissue profile (namely decreased levels of DHA and increased the lipoperoxides) and COX-2 levels in the rat frontal cortex. In addition, we found that BCCAO/R increased plasmatic levels of anandamide and lipoperoxides. The molecular changes induced by the BCCAO/R are evaluated on the basis of a single time point of reperfusion and, so far, this aspect represents an intrinsic limitation. Additional studies are warranted to evaluate both the time course of these changes during longer time points of reperfusion (e.g., at 6, 12 and 24 h after BCCAO/R) and the possible effects of dietary compounds in preventing BCCAO/R-induced oxidative stress.

In conclusion this study shows that BCCAO/R-induced positive modulation of the ECS. As far as we aware, this is the first study that has investigated early changes that can be easily traced in brain tissue as well as in plasma, and may be interpreted as indicative of the tissue physiological response to the oxidative stress induced by the BCCAO/R. The variations observed suggest that the activation of the ECS and the increase of pro-inflammatory substances are events that may be directly correlated.

## Abbreviations

AEA, anandamide: N-arachidonoyl-ethanolamide
2-AG: 2-arachidonoyl-glycerol
BCCAO/R: Bilateral common carotid artery occlusion/reperfusion
COX-2: Cyclooxygenase-2
DHA: Docosahexaenoic acid
eCBs: Endocannabinoids
ECS: Endocannabinoid system
GAPDH: Glyceraldehyde phosphate dehydrogenase
NAE: N-acylethanolamides
OEA: Oleoylethanolamide
PB: Phosphate buffer
PBS: Phosphate buffered saline
PEA: Palmitoylethanolamide
PPAR: Peroxisome-proliferator activated receptor
PUFA: Polyunsaturated fatty acids
SDS: Sodium dodecyl sulfate
TBS-T: Tris-buffered saline-Tween 20
